# Multilevel Deficiency of White Matter Connectivity Networks in Alzheimer's Disease: A Diffusion MRI Study with DTI and HARDI Models

**DOI:** 10.1155/2016/2947136

**Published:** 2016-01-13

**Authors:** Tao Wang, Feng Shi, Yan Jin, Pew-Thian Yap, Chong-Yaw Wee, Jianye Zhang, Cece Yang, Xia Li, Shifu Xiao, Dinggang Shen

**Affiliations:** ^1^Department of Geriatric Psychiatry, Shanghai Mental Health Center, Shanghai Jiao Tong University School of Medicine, Shanghai, China; ^2^IDEA Lab, Department of Radiology and BRIC, University of North Carolina at Chapel Hill, Chapel Hill, NC, USA; ^3^Alzheimer's Disease and Related Disorders Center, Shanghai Jiao Tong University, Shanghai, China; ^4^Department of Radiology, Shanghai Mental Health Center, Shanghai Jiao Tong University School of Medicine, Shanghai, China; ^5^Department of Brain and Cognitive Engineering, Korea University, Seoul, Republic of Korea

## Abstract

Alzheimer's disease (AD) is the most common form of dementia in elderly people. It is an irreversible and progressive brain disease. In this paper, we utilized diffusion-weighted imaging (DWI) to detect abnormal topological organization of white matter (WM) structural networks. We compared the differences between WM connectivity characteristics at global, regional, and local levels in 26 patients with probable AD and 16 normal control (NC) elderly subjects, using connectivity networks constructed with the diffusion tensor imaging (DTI) model and the high angular resolution diffusion imaging (HARDI) model, respectively. At the global level, we found that the WM structural networks of both AD and NC groups had a small-world topology; however, the AD group showed a significant decrease in both global and local efficiency, but an increase in clustering coefficient and the average shortest path length. We further found that the AD patients had significantly decreased nodal efficiency at the regional level, as well as weaker connections in multiple local cortical and subcortical regions, such as precuneus, temporal lobe, hippocampus, and thalamus. The HARDI model was found to be more advantageous than the DTI model, as it was more sensitive to the deficiencies in AD at all of the three levels.

## 1. Introduction

Alzheimer's disease (AD) is the most common form of dementia in elderly people and is characterized by chronic cortical atrophy and neurodegeneration, resulting in behavioral changes, loss of memory and language function, and general cognitive decline [1]. It is an irreversible and progressive brain disease and usually diagnosed in people older than 65. Nearly 36 million people worldwide are affected by AD, with 5.2 million alone just in the United States [2].

Tau and amyloid beta (A*β*) in cerebrospinal fluid are considered to be reliable biomarkers of AD [3]. However, the invasiveness, cost, and availability associated with the measurement of these quantities are significant drawbacks. On the other hand, magnetic resonance imaging (MRI) has been widely recognized as a noninvasive means for neurodiagnosis and disease staging. Previous studies using T1-weighted structural MRI revealed AD-induced gray matter (GM) atrophy in multiple brain regions, including the hippocampal and entorhinal cortices [4, 5], the temporal and cingulate gyri, the precunei, the insular cortices, the caudate nuclei, the frontal cortices [6], the sensorimotor cortices, the occipital poles, the cerebellum, and the medial thalami [7].

On the other hand, diffusion-weighted magnetic resonance imaging (DWI) [8] can recover the local profile of water diffusion in tissue, yielding information on white matter (WM) integrity and connectivity that is not available from standard structural MRI. Specifically, tractography methods [9, 10] can be used to fit continuous streamlines through directional diffusion data at each voxel for reconstructing WM fiber tracts. With the obtained tractography, WM integrity can be analyzed with both region-of-interest- (ROI-) based analysis, for example, tract-based spatial statistics (TBSS) [11] and fiber clustering [12, 13], and parcellation-based connectome analysis [14].

WM abnormalities in AD were reported in previous studies. Liu et al. [15] performed voxelwise TBSS to compare fractional anisotropy (FA) between the AD patients and the healthy controls. Multiple WM tracts, such as parahippocampal WM, cingulum, uncinate fasciculus, inferior and superior longitudinal fasciculus, and corpus callosum, showed decreased FA in the AD group. Jin et al. [16] used the tract-based clustering method to relate fornix degeneration to cognitive decline in AD with various diffusion-derived measures. Mean diffusivity (MD) was shown to be more sensitive to the group difference among AD and normal controls than FA did. Li et al. [17] proposed a spectral diffusional connectivity framework to explore the connectivity deficit in AD. The framework was based on studying eigenvalues of the Laplacian matrix of the diffusion tensor field at the voxel level. The peaks of the diffusional connectivity spectra were shifted in the AD group compared to the normal controls that did not shift. Daianu et al. [18] found widespread breakdown in AD in the 68-ROI based connectivity networks with multiple connectivity metrics on the “*k*-core” structure.

Importantly, WM tracts can be used to form the connectivity networks that give comprehensive pictures of interactions between different brain regions. A WM connectivity network can be described mathematically as a graph consisting of (1) a collection of nodes, representing the ROIs and (2) a set of edges between nodes, describing the connections (e.g., fiber counts) between ROI pairs. The characteristics of a connectivity network can be described using metrics at three hierarchical levels: global, regional, and local.

The stability of connectivity networks is influenced by multiple factors, including field strength [19, 20], scanners [21], imaging acquisition parameters [22], and tractography parameters [23]. Zhan et al. [24] compared several tractography and feature extraction methods in relation to AD diagnostic accuracy. Among these factors, the choice of diffusion models is found to be the most influential. The most commonly used approach, namely, diffusion tensor imaging (DTI), is based on the Gaussian assumption of water diffusion. This approach works well in regions with unidirectional fiber bundles, but this model may fail in regions with fiber crossings, which may introduce tractography errors in these regions. To address this issue, advanced models for high angular resolution diffusion imaging (HARDI) were proposed to estimate orientation distribution functions (ODF) [25, 26] at each voxel. By detecting the peaks (i.e., local maxima) of the ODF, one can then infer the number of directions contained in each voxel.

In this study, we investigate the global, regional, and local changes of whole-brain connectivity networks in AD patients in comparison to healthy elderly subjects. DTI and HARDI models are used to construct two different sets of connectivity networks for comparison. Such a systematic network analysis at multiple levels on AD, to our knowledge, has not been attempted previously.

## 2. Methods

### 2.1. Participant Recruitment

This study involved 26 patients who were diagnosed with probable AD at the Alzheimer's Disease and Related Disorders Center (ADRDC) in the tertiary hospital of Shanghai Mental Health Center (SMHC) at Shanghai Jiao Tong University School of Medicine. 16 cognitively healthy elderly subjects from the community of Shanghai Chang Ning district were included as the normal control (NC) group. Subjects were enrolled via self-referral or referral from families or physicians. The study was conducted from May 2011 to May 2012 at ADRDC. The SMHC Institution's Ethical Committee approved the consent form and the study protocol. The study was carried out in accordance with the Declaration of Helsinki. Informed consent was obtained from all subjects and/or their legal guardians.

The ages of the AD subjects enrolled ranged from 50s to 90s. Prior to enrollment, patients provided their medical history and were given physical and neurological examinations, laboratory tests, and both T1-weighted and fluid-attenuated inversion recovery (FLAIR) MRI scans. Enrollment criteria included (1) the National Institute of Neurological and Communicative Disorders and Stroke/Alzheimer's Disease and Related Disorder Association (NINCDS/ADRDA) criteria for probable AD [27]; (2) the Diagnostic and Statistical Manual for Mental Disorders, 4th edition (DSM-IV) criteria for the Alzheimer's dementia; (3) a Hachinski Ischemia Score less than 4; (4) the systolic blood pressure between 95 and 160 and the diastolic blood pressure between 60 and 95; (5) identification of a responsible and consistent caregiver; (6) absence of diabetes, renal impairment, significant systemic conditions, psychiatric disorders, seizures, or traumatic brain injuries that could compromise their cognitive or brain functions; (7) significant brain abnormalities on the patient's T1-weighted MRI; (8) clinical score requirements. In the Chinese version of the Mini-Mental Status Exam (MMSE) [28], there are three cut-off thresholds for AD diagnosis according to education levels: (1) AD subjects who had not been educated exhibited MMSE scores <18; (2) those with elementary school education exhibited MMSE scores <21; (3) those with higher than middle school education exhibited MMSE scores <25. The Clinical Dementia Rating (CDR) scale [29] was equal or more than 1.

The NC group was cognitively functioning healthy individuals. The NC group did not have any history of cognitive decline, neurological disorders, or uncontrolled systemic medical disorders. Their CDR scales were equal to 0. All subjects in the study were required to have fewer than two lacunar ischemia strokes (of diameter <1 cm) in the brain, as revealed by FLAIR.

The demography and clinical scores for the AD group and the NC group are listed in Table 1. No significant differences between the two groups were observed in age or education. The difference was observed in gender. However, the effects of gender, age, education level, and brain size would be regressed out in our analysis. As expected, the group difference was observed in the MMSE (*p* < 0.001) and CDR scores (*p* < 0.001).

### 2.2. MR Image Acquisition

MRI images were scanned with a Siemens MAGNETOM VERIO 3 T scanner at SMHC. T1-weighted images were obtained with 128 sagittal slices using the 3D magnetization prepared rapid acquisition gradient echo (MPRAGE) sequence with the following parameters: TR = 2,530 ms, TE = 3.39 ms, flip angle = 7°, and spatial resolution = 1 × 1 × 1.3 mm^3^, and the acquisition time was 8 minutes 7 seconds. The DWI images were acquired with 75 axial slices by using an echo planar imaging (EPI) sequence that covered the whole brain. The acquisition parameters were as follows: TR = 10,000 ms, TE = 91 ms, and spatial resolution = 2 × 2 × 2 mm^3^. A total volume of 62 directions was acquired, where two volumes were without diffusion gradient (*b* = 0) and the rest 60 volumes were with diffusion gradient of *b* = 1,000 s/mm^2^. The acquisition time was 5 minutes and 42 seconds.

### 2.3. Image Preprocessing

T1-weighted images were first resampled to be 1 mm isotropic, intensity inhomogeneity corrected [30], and skull stripped to remove nonbrain tissues [31]. The resulting images were then tissue segmented to separate GM, WM, and cerebrospinal fluid (CSF) with FSL FAST (http://fsl.fmrib.ox.ac.uk/fsl/fslwiki/). DWI images were skull stripped and then corrected for eddy-current induced distortion with FSL. FA and MD images were then extracted from DWI data after diffusion tensor fitting.

### 2.4. Network Construction

Whole-brain tractography was performed with both DTI and HARDI models. For DTI, the diffusion tensors were fitted to the data using a weighted-least-squares estimation algorithm and the eigenvector of the largest eigenvalue was taken as the principal diffusion direction [32]. Seed points were chosen as voxels with FA > 0.3. The maximum turning angle was set to 45° and tracking was stopped when FA < 0.2. The allowed fiber length was at minimum, 20 mm, and at maximum, 300 mm. For HARDI, ODFs were estimated with dictionary-based spherical deconvolution [26]. A maximum of three peaks were detected from the ODF at each voxel [26]. Four seeds were randomly sampled within each seed voxel. The criteria of fiber tracking were the same for both methods. The resulting tractography was manually visualized and checked in ParaView (Kitware, http://www.paraview.org/).

The Automated Anatomical Labeling (AAL) template [33] is a widely used high-resolution T1-weighted brain parcellation based on a single adult subject, which includes 90 cortical and subcortical brain regions for the cerebrum. The names and abbreviations of these 90 ROIs are listed in Table 2. First, we nonlinearly registered the AAL template to each subject's segmented T1-weighted image using HAMMER [34]. Then, the T1-weighted image was rigidly aligned to the FA image. The original 90 ROIs of the AAL template were transferred to each individual's DWI space using the deformation fields and the affine transformation matrix generated during the registration step. These ROIs were used as nodes in the connectivity network for each subject.

Two ROIs were considered anatomically connected, if there were fibers traversing them. In the network, the edge, connecting the nodes representing these two ROIs, was defined as the number of fibers connecting them. Two ROIs were considered connected if there were no less than four fibers between them, which has been proven effective to reduce false-positive connections [35–37]. As a result, the WM connectivity network, represented by a symmetric 90 × 90 matrix, was constructed for each subject. The network was weighted and undirected.

### 2.5. Multilevel Network Measures

Three hierarchical levels (global, regional, and local) of complex network measures were used to compare the measures of connectivity networks constructed in Section 2.4 between the AD group and the NC group. The measures were calculated with the GRETNA toolbox (https://www.nitrc.org/projects/gretna/). For a detailed review of those measures, please see [38].

#### 2.5.1. Global Measures

Global and local network efficiencies are used to describe global and local characteristics of parallel information transfer in a network. Global network efficiency quantifies the exchange of information across the entire brain:

(1) $$\begin{array}{c} E_{glob}=\frac{1}{N\left(N-1\right)}\overset{N}{\underset{i=1}{\sum }}\;\overset{N}{\underset{j=1,j\neq i}{\sum }}\frac{1}{L_{ij}}, \end{array}$$

where *L*_*ij*_ is the shortest absolute path length between node *i* and node *j*. *N* is the total number of nodes. Similarly, local network efficiency of node *i* is defined as

(2) $$\begin{array}{c} E_{loc}^{i}=\frac{1}{N_{G_{i}}\left(N_{G_{i}}-1\right)}\overset{N_{G_{i}}}{\underset{j=1}{\sum }}\;\overset{N_{G_{i}}}{\underset{k=1,k\neq j}{\sum }}\frac{1}{L_{jk}}, \end{array}$$

where *G*_*i*_ is a subgraph comprising nodes directly connected to node *i*, and *N*_*G*_*i*__ is the node number of the subgraph *G*_*i*_. Therefore, the average local network efficiency for the whole brain is *E*_loc_ = (1/*N*)∑_*i*=1_^*N*^ *E*_loc_^*i*^.

The global clustering coefficient gives an overall indication of clustering in a network. It is the average of absolute local clustering coefficients of all nodes:

(3) C=1N∑i=1NCi,Ci=EiKiKi−1/2,

where *C*_*i*_ is the local clustering coefficient for node *i*, *E*_*i*_ is the number of edges in the subgraph *G*_*i*_ of node *i*, and *K*_*i*_ denotes the number of nodes in *G*_*i*_. In other words, *C*_*i*_ is the proportion of edges between the nodes within the neighborhood of node *i* divided by the number of edges that could possibly exist between them. In addition, the average shortest path length of the network is defined as

(4) $$\begin{array}{c} L=\frac{1}{N\left(N-1\right)}\overset{N}{\underset{i=1}{\sum }}\;\overset{N}{\underset{j=1,j\neq i}{\sum }}L_{ij}. \end{array}$$

The human brain exhibits the “small-world” property characterized by dense local clustering between neighboring nodes and high global network efficiency with short path lengths due to the existence of relatively few long-range connections [39–41]. Mathematically, it can be represented by the ratio of the normalized global clustering coefficient *γ* = *C* ^real^/*C* ^rand^ to the normalized shortest path length *λ* = *L* ^real^/*L* ^rand^, where *C* ^rand^ and *L* ^rand^ are the global clustering coefficient and the normalized shortest path length of a random network. A random network was simulated by iteratively rewiring 50% pairs of random edges of the existing brain network for 1,000 times. Then, small-worldness can be measured as *σ* = *γ*/*λ* [42] and it should be greater than 1 if the graph demonstrates the small-world property.

#### 2.5.2. Regional Measures

The nodal efficiency was computed to represent the regional characteristics of a network. The nodal efficiency *E*_*i*_ is defined as

(5) $$\begin{array}{c} E_{i}=\frac{1}{N-1}\overset{N}{\underset{j=1,j\neq i}{\sum }}\frac{1}{L_{ij}}, \end{array}$$

where *L*_*ij*_ is the shortest path length between node *i* and node *j*. Therefore, *E*_*i*_ measures the overall information flow between a given node *i* and the rest of the nodes in the network. The node *i* is defined as a hub if *E*_*i*_ is at least 1 standard deviation (SD) above the average nodal efficiency of the network.

#### 2.5.3. Local Measures

The network edges, that is, the fiber counts between each pair of ROIs, were directly used to describe the local characteristics of a network.

### 2.6. Statistical Analysis

The nonparametric permutation test was used to evaluate statistical differences of brain network properties between the AD and NC groups. First, linear regression was performed on all the network measures at global, regional, and local levels (described in Section 2.5), respectively, with age, gender, level of education, and intracranial volume (ICV) as covariates. Then, after removing those factors on the measures, the regressed measures were permuted 5,000 times to assess the statistical significance of the differences [36]. The significance level was set as *p* < 0.05, with false discovery rate (FDR) [43] for multiple comparison correction. To compare the performance between the DTI and HARDI methods, the same analysis was performed to the networks constructed by each method, respectively.

## 3. Results

### 3.1. DTI versus HARDI

**Figure 1:**
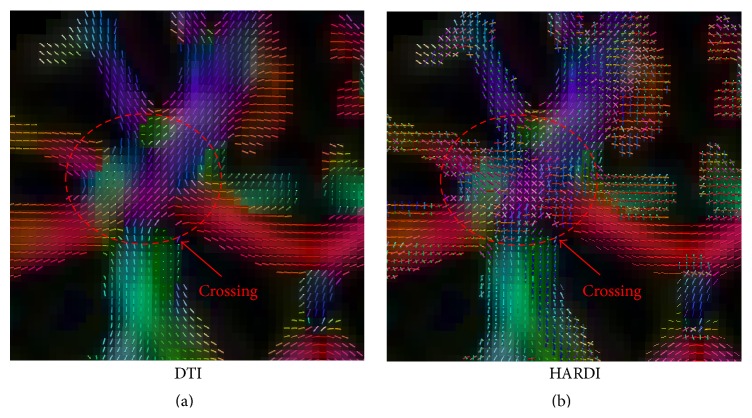
Directional glyphs at the intersection of the left corticospinal tract and the corpus callosum given by (a) the DTI method and (b) the HARDI method.

**Figure 2:**
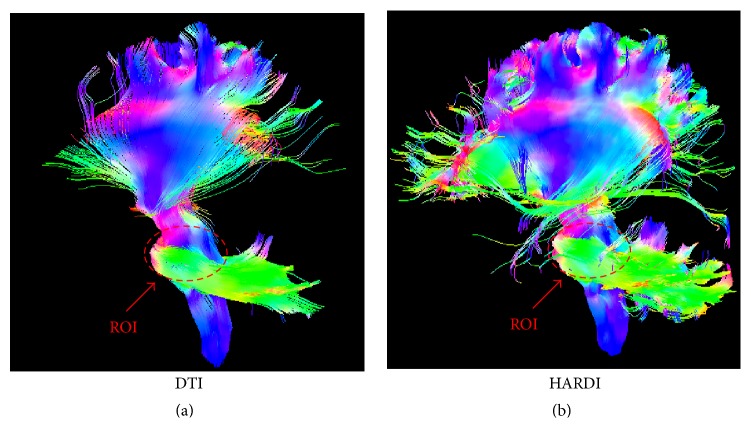
Tractography results based on a seed ROI at the brain stem with (a) the DTI method and (b) the HARDI method.

We compared the DTI and HARDI networks, in terms of their ability, to distinguish the AD group from the NC group.  Figure 1 shows that HARDI method can handle fiber crossings in the intersection between the left corticospinal tract and the corpus callosum. The DTI method, on the other hand, was not able to do so.  Figure 2 shows the tractography results based on a seed ROI near the brain stem. The HARDI method was able to produce significantly more fibers than the DTI method.

**Figure 3:**
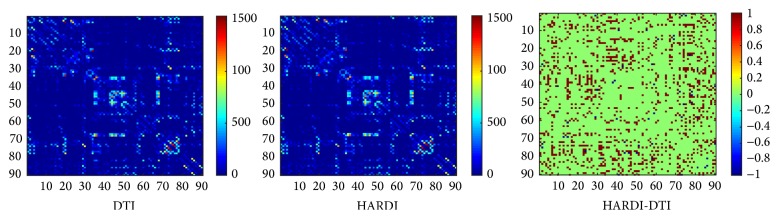
The 90 × 90 connectivity matrices built with the DTI method and the HARDI method, respectively. The right panel shows the binary difference between the left two matrices for a selected subject, where the entries with +1 denote connections detected by HARDI but not DTI, and −1 for connections detected by DTI but not HARDI.

Figure 3 shows the 90 × 90 connectivity matrices (≥4 fiber connections) with both DTI and HARDI methods from a randomly selected subject in our dataset. The binary difference between the two matrices is also shown, where the entries with +1 denote connections detected by HARDI but not DTI, and −1 for connections detected by DTI but not HARDI. For this selected subject, the meaningful connections (≥4 fiber connections) account for 38% and 52% out of the total connections for DTI and HARDI, respectively. From the difference map in the right panel of Figure 3, it is obvious that more connections can be detected with HARDI compared to DTI.

### 3.2. Global Characteristics

Both the NC and the AD groups showed small-world organization (*σ* > 1) in their networks. The AD networks actually showed higher small-worldness than the NC networks did, in both DTI and HARDI cases (*σ*_AD_ > *σ*_NC_). In both cases, the AD group, when compared to the NC group, showed decreases in global efficiency and local efficiency but increases in the normalized shortest path length (*λ*) and the normalized clustering coefficient (*γ*). Also, all results given by the HARDI method were statistically significant (*p* < 0.05), while most results by the DTI method were not, except global efficiency.  Table 3 lists the values of these measures for the AD and NC groups by both the DTI and HARDI methods.

### 3.3. Regional Characteristics

**Figure 4:**
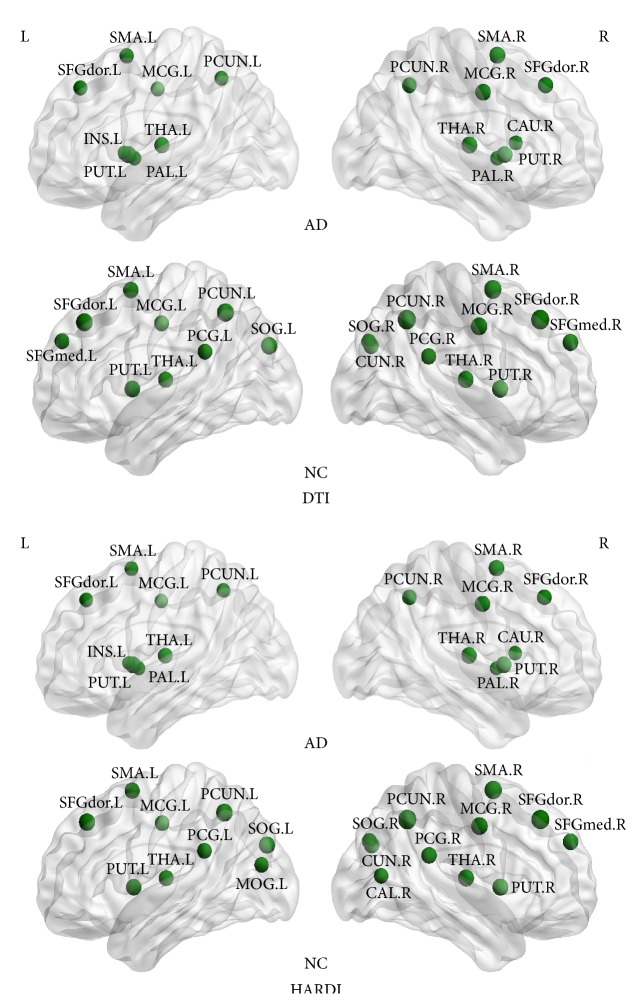
Hubs in the AD group and the NC group given by the DTI method and the HARDI method, respectively. Each sphere represents the center of an ROI. Refer to Table 2 for the label of each ROI.

An ROI is defined as a network hub, if its nodal efficiency is 1 SD greater than the mean nodal efficiency of the network. For the HARDI case, we identified 20 hub nodes in the NC group, including 6 association cortical regions, 13 paralimbic cortical regions, and 1 primary cortical region. Only 16 hub nodes were identified in the AD group, including 5 association regions and 11 paralimbic regions. In both groups, 12 ROIs were identified as hubs in common, including the bilateral superior frontal gyri, dorsolateral (SFGdor), the bilateral supplementary motor areas (SMA), the bilateral median cingulate gyri (MCG), the bilateral precunei (PCUN), the bilateral putamina (PUT), and the bilateral thalami (THA). In addition, 4 ROIs, such as the left insula (INS.L), the right caudate nucleus (CAU.R), and the bilateral pallida (PAL), were identified as the hubs in the AD group but not in the NC group. 8 ROIs, such as the right medial superior frontal gyrus (SFGmed.R), the bilateral posterior cingulate gyri (PCG), the right calcarine cortex (CAL.R), the right cuneus (CUN.R), the bilateral superior occipital gyri (SOG), and the left middle occipital gyrus (MOG.L), were identified as the hubs in the NC group but not in the AD group. For the DTI case, most of the hubs identified in the HARDI case were also detected. The AD group had the exact 16 hubs as in the HARDI case, while the NC group only had 19 hubs. The right calcarine cortex (CAL.R) and the left middle occipital gyrus (MOG.L) were only identified in the HARDI case for the NC group, while the left medial superior frontal gyrus (SFGmed.L) was only identified in the DTI case. The hub distributions in the AD and NC groups are shown in Figure 4 for both methods.

**Figure 5:**
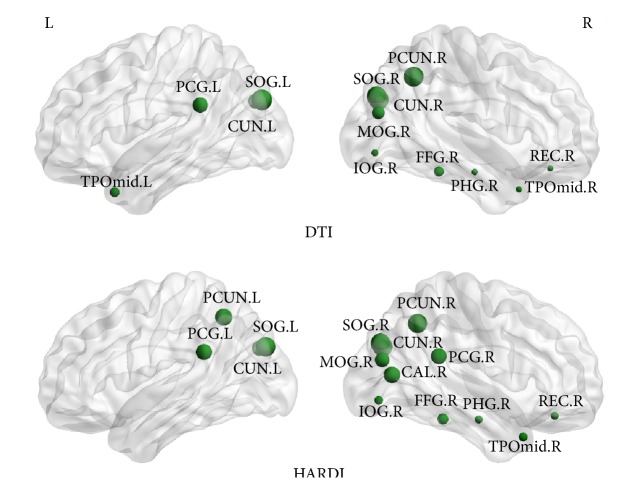
ROIs that have the reduced nodal efficiency in the AD group compared to the NC group (*p* < 0.05), given by the DTI and HARDI methods, respectively. Each sphere represents the center of an ROI and its size is proportional to the nodal efficiency. Refer to Table 2 for the label of each ROI.

In both DTI and HARDI cases, when compared to the NC group, the AD group showed reduced nodal efficiency (*p* < 0.05, FDR corrected) in the bilateral superior occipital gyri (SOG), the right middle occipital gyrus (MOG.R), the right rectus gyrus (REC.R), the left posterior cingulate gyrus (PCG.L), the right parahippocampal gyrus (PHG.R), the right middle temporal pole (TPOmid.R), the right inferior occipital gyrus (IOG.R), the right fusiform gyrus (FFG.R), the right precuneus (PCUN.R), and the bilateral cunei (CUN). Besides all of the regions shown above, the right posterior cingulate gyrus (PCG.R), the right calcarine cortex (CAL.R), and the left precuneus (PCUN.L) showed the significantly reduced nodal efficiency only in the HARDI case, while the left middle temporal pole (TPOmid.L) showed the reduced efficiency only in the DTI. The comparison between the ROIs that had the reduced efficiency in the two groups for the DTI and HARDI cases is shown in Figure 5.

### 3.4. Local Characteristics

**Figure 6:**
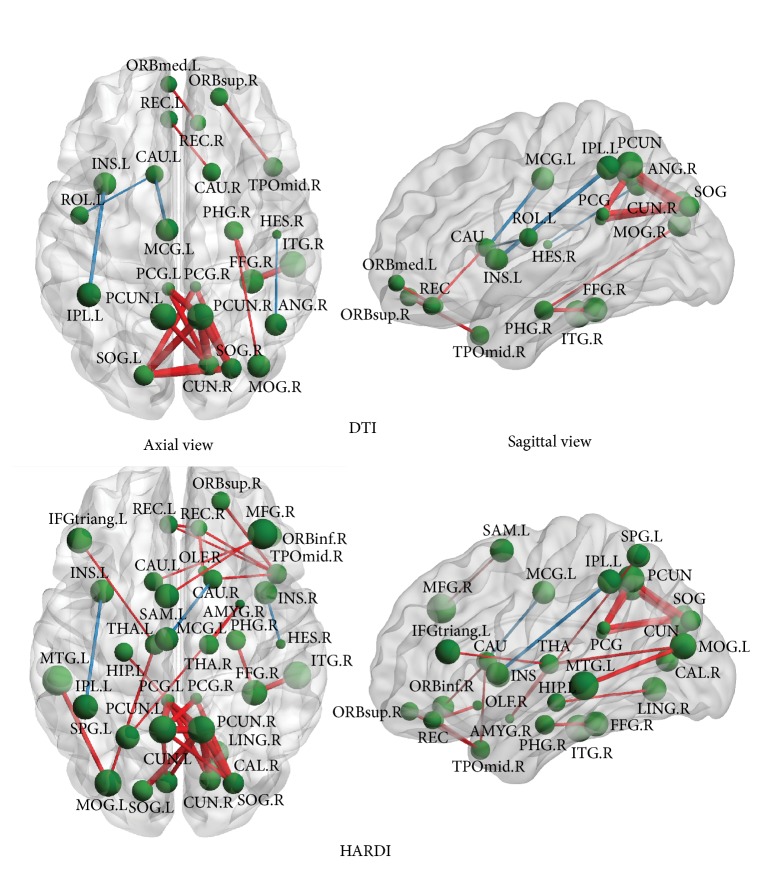
The axial and the sagittal views of the significantly different connections (*p* < 0.05) based on the fiber counts between the AD group and the NC group given by the DTI and HARDI methods, respectively. The stronger connections (higher fiber counts between a pair of ROIs) in the AD group are shown in* blue*, while the weaker connections (lower fiber counts between a pair of ROIs) are in* red*. Refer to Table 2 for the label of each ROI.

**Figure 7:**
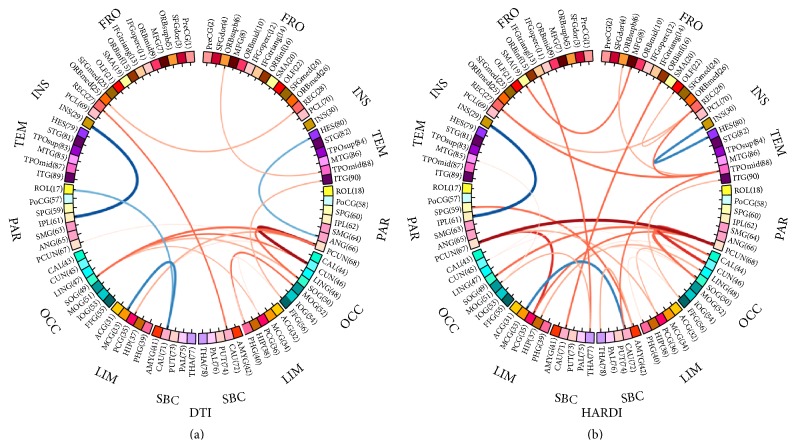
Connectograms showing significantly different connections (*p* < 0.05) based on the fiber counts between the AD group and the NC group, given (a) the DTI method and (b) the HARDI method. The thickness of each line indicates the extent of the difference between the corresponding connections in the two groups. The stronger connections (higher fiber counts between a pair of ROIs) in the AD group are shown in* blue*, while the weaker connections (lower fiber counts between a pair of ROIs) are in* red*. Refer to Table 2 for the label of each ROI.

We utilized the fiber counts between a pair of ROIs to measure the strength of their connection. After performing the permutation test [36] on each connection, the axial and the sagittal views of those significantly different connections (*p* < 0.05) between the AD group and the NC group, with the DTI and HARDI method, are illustrated in Figure 6. Additionally, the connectogram, a circular representation tool called Circos (http://www.cpan.org/ports/) [44], was used to demonstrate those connections with the two models in Figure 7. In both figures, the stronger connections (higher fiber counts between a pair of ROIs) in the AD group are shown in* blue,* and the weaker connections (lower fiber counts between a pair of ROIs) are in* red*. Particularly, the thicker the line in Figure 7, the greater the difference in the connection between the two groups. The identified differences in connections spread over the entire brain. A large portion of these differences was located in the limbic system and subcortical regions. It is obvious that the HARDI model was able to detect noticeably more pairs of different connections between the groups (30 pairs in HARDI versus 20 pairs in DTI). For example, the connections through the left supplementary motor area (SMA.L), the right lingual gyrus (LING.R), the left superior parietal gyrus (SPG.L), the bilateral thalami (THA), the left middle temporal gyrus (MTG.L), and the left hippocampus (HIP.L) were only shown in the HARDI case.

## 4. Discussion

This study investigates the impact of AD on the topological characteristics of the WM connectivity network at three hierarchical levels, global, regional, and local level, through tractography data reconstructed using DTI and HARDI methods, respectively. The main findings are as follows: (1) the global and average local network efficiency are reduced in AD, with increased shortest path length; (2) the number of regional hubs and nodal efficiency decreases in AD; (3) the local connections weaken in AD; (4) the HARDI method has an advantage over the DTI method in identifying more abnormal network characteristics at all the three levels.

At the global level, consistent with the previous studies [41, 45, 46], our results indicate that the WM connectivity networks of both AD and NC have the small-world topology. Although the AD networks show a slightly elevated small-world attribute, most global measures are lower in AD, compared to those in NC. AD patients show significant decreases in global efficiency and average local efficiency, but increases in normalized shortest path length. Global efficiency and average local efficiency are known to reflect the overall ability of information transfer between different nodes in a network. They are comprehensive indices for the capability of parallel information processing. The reductions in those measures can be attributed to the degeneration of WM, which indicates that connections between cortical regions are abnormal. The less strength of connections between cortical regions is due to the damaged WM integrity, resulting in longer pathways that connect different regions in the brain. The breakdown in the optimal brain balance between the local specialization and the global integration causes information processing to malfunction in AD. Similarly to previous studies [47], we have also found that the normalized weighted shortest path length increases in the AD group. Shortest path length ensures interregional effective communication, or prompt transfer of information between regions, which constitutes the basis of cognitive processes [48]. The WM damage can lead to an increase in shortest path length. Therefore, it is likely that, in people with AD, information may flash in a certain brain region but fail to transmit to other regions effectively to form stable memories. The normalized clustering coefficient is a ratio of local information transfer capability in a network to that of a random network. In AD, its increase reflects the reinforcement of information transfer capability. Likewise, previous studies have also found a greater clustering coefficient and a longer absolute path length in AD, which may indicate that the organization of the cortical network is least optimal in AD [49].

At the regional level, several hubs identified in NC are not shown in AD, such as precuneus (PCUN) and posterior cingulate gyrus (PCG). These two regions also demonstrate reduced connectivity in functional magnetic resonance imaging (fMRI) studies in patients with amnesic mild cognitive impairment (aMCI), a stage with high risk in developing AD [50], which may suggest that these two regions maintain pivotal roles in both structural and functional default mode networks in AD [51].

The AD networks also show decreased nodal efficiency in many cortical regions, mainly located in the bilateral cunei (CUN), the right precuneus (PCUN.R), the bilateral posterior cingulate gyri (PCG), the right temporal pole, middle (TPOmid.R), and the right parahippocampal gyrus (PHG.R). The cuneus (CUN), the precuneus (PCUN), and the posterior cingulate gyrus (PCG) are thought to be involved in the episodic memory information transmission and malfunction in AD [52]. Although the degeneration of the posterior cingulate gyrus was originally interpreted as not being a direct consequence of degeneration in the medial temporal lobe, recent studies have revealed that this area has atrophy and metabolic abnormalities in incipient AD [52–54]. In a study that examined the cingulum tract in AD, both the anterior and posterior regions were affected [55]. The posterior cingulate region is a key “hub” affected in AD. The temporal pole, middle (TPOmid), and the parahippocampal gyrus (PHG) are involved in semantic memory processing and recognition [56] and show atrophy and neuronal loss in AD [57, 58]. Notably, the decreased efficiency in the temporal lobes was observed to be mainly located in the right hemisphere. Together, the reduced nodal efficiency suggests that possible WM degeneration in these brain regions may negatively affect information transmission and functional integration in AD patients.

At the local level, weaker connection (lower fiber counts) happens predominantly in the area of the bilateral precunei (PCUN), the right cuneus (CUN.R), the left middle temporal gyrus (MTG.L), and left hippocampal gyrus (HIP.L). These areas, which are mostly associated with linguistic integration, emotion, and semantic memory [56, 59], are affected in AD patients [57, 58]. It is worth noting that precuneus, cuneus, and temporal lobe also show reduced nodal efficiency at the regional level. In addition to the typically well-known affected regions in AD, the right amygdala (AMYG.R) and the right middle frontal gyrus (MFG.R) show weaker connections as well. These regions are the structures mostly involved in emotional processing, perceptions, psychological states, and behavioral responses [60]. Weaker connections can also be identified at the right thalamus (THA.R), which is known to have a significantly reduced volume in AD [61]. Interestingly, a few regions show increased local connection in the AD group than in the NC group, for example, the connection between the left insula (INS.L) and left inferior parietal lobule (IPL.L). It is possible that this may result from the compensation to weak connections in the neighboring regions.

Overall, the HARDI method outperforms the DTI method in terms of differentiating AD and NC at all three levels. At the global level, the HARDI method has more statistical power in distinguishing the groups for all the measures, according to the *p* values in Table 3. The group differences of all the measures are statistically significant in the HARDI case, while most of them are not statistically significant in the DTI case. At the regional level, the HARDI method detects more regions with reduced nodal efficiency. These include the bilateral posterior cingulate gyri (PCG) and the bilateral precunei (PCUN), while the results of the DTI method only show the unilateral deficiency of these regions. The results from the HARDI method are more consistent with the pathology of AD, as the bilateral posterior cingulate gyri and precunei are both associated with memory processing and affected in AD [62, 63]. At the local level, the two methods show the greatest difference. The HARDI method is able to identify 50% more of the weaker connections in AD than the DTI method (30 pairs versus 20 pairs). This may be because the HARDI method is able to find the correct tract directions at the fiber crossing regions and can find more connections in the NC group. Specifically, the left superior parietal gyrus (SPG.L), the right thalamus (THA.R), and the left middle temporal gyrus (MTG.L), especially the left hippocampus (HIP.L) and the left cuneus (CUN.L), are only found using the HARDI method. During the early onset of AD, the superior parietal gyrus and the middle temporal gyrus undergo neuronal loss [64]. Besides the neocortical atrophy, subcortical structures, such as the thalamus, also suffer atrophy and may contribute to cognitive decline and emotion disorder in AD [65].

Fiber count is one of the most commonly used measures in evaluating connectivity characteristics. For example, Dennis et al. [66] computed graph theory metrics based on the fiber count to track changes in both structural connectivity and network efficiency in young healthy individuals, while Zhan et al. [67] developed a machine learning framework to classify different stages of AD with fiber counts as features. However, sometimes, fiber count may not be a suitable feature in connectivity studies. For instance, in [68, 69], the networks constructed with the mean FA, MD, and fiber length provided better performance in identifying high-risk autistic infants than fiber count. Therefore, we will consider incorporating other network measures in our future work since they may provide additional insights into connectivity breakdown, especially for the case that the fiber count based networks cannot reveal the progression of AD.

The conventional statistical analysis on network properties is often performed in a univariate manner, that is, pairwise comparison between groups. This might overlook the interaction among sets of connections in group difference. On the other hand, instead of doing simple pairwise comparison, a classification framework is able to consider all individual connection features, as well as their relationships, for selecting the most discriminative features for classification [68–70]. Ensemble learning algorithm, such as random forest, is one of this type of classification algorithms that can be applied to identify discriminative connectivity patterns in a multivariate manner for AD or MCI classification. This will be our future work.

We do acknowledge that there are some limitations in this study. Firstly, the sample size of our study is quite small. In the future, more participants need to be recruited to increase the statistic power of the results. Secondly, the lack of gold standard for regional parcellation makes the definition of ROI not very precise, especially on the boundary. Registration error may also play a role in this issue. Therefore, it may affect the accuracy in the analysis of connectivity networks [71, 72]. Thirdly, the underlying biological relationship between the network properties and the AD progression is currently unclear. Studying the intermediate stage, for example, MCI, may be beneficial for further understanding of the relationship [73, 74]. In the future work, we will include participants from this stage to perform a more comprehensive study on this topic.

## 5. Conclusion

In summary, we performed a systematic study on the WM connectivity comparison at three hierarchical levels (global, regional, and local) between the two groups: the AD group and the NC group. The analysis was conducted using tractography data generated using two diffusion models (DTI and HARDI) to evaluate the influence of tractography on the network analysis. Globally, both the AD group and the NC group demonstrate the small-world topology. However, many global measures, such as global efficiency, average local efficiency, and normalized shortest path length, were suboptimal in the AD group. Regionally, the AD group had the reduced number of hubs and significantly decreased nodal efficiency in the precuneus and the temporal lobe (the well-known atrophic regions in AD). Locally, weaker connections exist in these regions, as well as regions in the limbic system and the subcortex, such as hippocampus and thalamus. The HARDI method outperforms the DTI method at all three levels since the advanced model in the HARDI method can more accurately reflect the underlying complex fiber configurations.

## Figures and Tables

**Table 1:** The demography and clinical scores of the subjects in the study. The *p* values are based on the two-sample *t*-tests except the gender. The gender ratio was examined by the Chi-squared test.

	NC (*n* = 16)	AD (*n* = 26)	*p* value
Age (years)	70.1 ± 7.5	69.5 ± 7.1	0.81
Male/female	11/5	8/18	0.03
Education (years)	10.6 ± 3.2	10.4 ± 3.9	0.91
CDR	0.0 ± 0.0	2.0 ± 0.7	<0.001
MMSE	25.3 ± 3.6	15.2 ± 6.5	<0.001

**Table 2:** Names and abbreviations of the 90 ROIs defined in the AAL template.

Index^*∗*^	Region	Abbreviation
1, 2	Precentral gyrus	PreCG
3, 4	Superior frontal gyrus (dorsal)	SFGdor
5, 6	Orbitofrontal cortex (superior)	ORBsupb
7, 8	Middle frontal gyrus	MFG
9, 10	Orbitofrontal cortex (middle)	ORBmid
11, 12	Inferior frontal gyrus (opercular)	IFGoperc
13, 14	Inferior frontal gyrus (triangular)	IFGtriang
15, 16	Orbitofrontal cortex (inferior)	ORBinf
17, 18	Rolandic operculum	ROL
19, 20	Supplementary motor area	SMA
21, 22	Olfactory	OLF
23, 24	Superior frontal gyrus (medial)	SFGmed
25, 26	Orbitofrontal cortex (medial)	ORBmed
27, 28	Rectus gyrus	REC
29, 30	Insula	INS
31, 32	Anterior cingulate gyrus	ACG
33, 34	Middle cingulate gyrus	MCG
35, 36	Posterior cingulate gyrus	PCG
37, 38	Hippocampus	HIP
39, 40	Parahippocampal gyrus	PHG
41, 42	Amygdala	AMYG
43, 44	Calcarine	CAL
45, 46	Cuneus	CUN
47, 48	Lingual gyrus	LING
49, 50	Superior occipital gyrus	SOG
51, 52	Middle occipital gyrus	MOG
53, 54	Inferior occipital gyrus	IOG
55, 56	Fusiform gyrus	FFG
57, 58	Postcentral gyrus	PoCG
59, 60	Superior parietal gyrus	SPG
61, 62	Inferior parietal lobule	IPL
63, 64	Supramarginal gyrus	SMG
65, 66	Angular gyrus	ANG
67, 68	Precuneus	PCUN
69, 70	Paracentral lobule	PCL
71, 72	Caudate	CAU
73, 74	Putamen	PUT
75, 76	Pallidum	PAL
77, 78	Thalamus	THA
79, 80	Heschl gyrus	HES
81, 82	Superior temporal gyrus	STG
83, 84	Temporal pole (superior)	TPOsup
85, 86	Middle temporal gyrus	MTG
87, 88	Temporal pole (middle)	TPOmid
89, 90	Inferior temporal	ITG

^*∗*^The odd and even indices indicate the regions in the left and right hemispheres, respectively.

**Table 3:** The comparison of the global connectivity characteristics between the AD and the NC groups with the DTI and HARDI models, respectively.

	*E*_glob_	*E*_loc_	*γ*	*λ*	*σ*
DTI					
AD	524 ± 93	674 ± 134	1.46 ± 0.18	1.20 ± 0.06	1.22 ± 0.11
NC	617 ± 114	748 ± 121	1.39 ± 0.12	1.16 ± 0.04	1.20 ± 0.07
Difference	−93	−74	0.07	0.03	0.02
*p* value	**0.03**	0.12	0.12	0.07	0.20
HARDI					
AD	426 ± 100	578 ± 126	1.66 ± 0.24	1.27 ± 0.08	1.31 ± 0.16
NC	543 ± 134	674 ± 144	1.53 ± 0.19	1.22 ± 0.06	1.25 ± 0.11
Difference	−116	−95	0.14	0.05	0.06
*p* value	**0.01**	**0.05**	**0.03**	**0.03**	**0.04**
